# Peritoneal Cancer Mimicking Sclerosing Mesenteritis: A Case Report

**DOI:** 10.7759/cureus.20934

**Published:** 2022-01-04

**Authors:** Naoto Mouri, Ryuichi Ohta, Chiaki Sano

**Affiliations:** 1 Community Care, Unnan City Hospital, Unnan, JPN; 2 Communiy Care, Unnan City Hospital, Unnan, JPN; 3 Community Medicine Management, Shimane University Faculty of Medicine, Izumo, JPN

**Keywords:** ca-125, exploratory laparoscopy, physical examination, history taking, rural hospital, sclerosing mesenteritis, peritoneal cancer

## Abstract

Peritoneal cancer is a rare disease that typically affects middle-aged women. Sclerosing mesenteritis can have a benign or malignant etiology. Although computed tomography (CT) scan and magnetic resonance imaging have been used to differentiate these two diseases, the findings are not always conclusive. Here, we report the case of an older woman who presented with acute abdominal pain. She was initially diagnosed with sclerosing mesenteritis, but the final diagnosis was peritoneal cancer. The initial treatment included antibiotics, non-steroidal anti-inflammatory drugs, and prednisolone. Tamoxifen was administered due to persistent symptoms, which were alleviated. However, the patient’s cancer antigen 125 levels were elevated, and there were changes in the peritoneal CT findings. The patient was diagnosed with primary peritoneal cancer based on further investigation of the peritoneum using positron emission tomography-CT and a biopsy. This case report describes the diagnostic process regarding the differentiation between sclerosing mesenteritis and primary peritoneal cancer when the CT findings mimic those of sclerosing mesenteritis in general medicine.

## Introduction

Sclerosing mesenteritis is a rare disease characterized by acute inflammation of the abdominal mesentery, causing abdominal pain with various complications, such as small bowel and urinary tract obstruction [1]. Its etiology varies from autoimmune to iatrogenic [1]. The differential diagnosis includes local and systemic diseases [2]. Radiologically, it presents as a soft tissue mass with fat ringing, tumor pseudo-capsule, vascular abnormalities, calcifications, and misty mesentery [3]. The presence of a soft tissue mass and tumor pseudo-capsule lesions warrants investigation for cancers and sarcoidosis. Diffuse inflammatory lesions are highly suggestive of metastasis or autoimmune diseases [4,5]. Another rare cause of peritonitis is peritoneal cancer [6]. This cancer is progressive and involves the gastrointestinal tract, ovaries, and bladder [7]. It typically presents with abdominal pain, ascites, and vague symptoms [6].

It is essential to differentiate sclerosing mesenteritis and peritoneal cancer because tamoxifen is effective against both diseases, but peritoneal cancer requires more intensive treatment [6]. The diagnosis of sclerosing mesenteritis is based on clinical findings because pathological findings, such as a mesenteric biopsy, do not aid in its diagnosis [1].

A computed tomography (CT) scan helps confirm the diagnosis and exclude other diseases such as peritoneal cancer [8]. A diffuse and misty mesentery on a CT scan suggests sclerosing mesenteritis. However, the imaging findings change depending on the clinical course of the disease [9]. Changes in the CT findings make the diagnosis and treatment of this disease challenging.

Here, we report a case of peritoneal cancer that mimicked sclerosing mesenteritis on abdominal CT imaging. Initially, a misty mesentery was detected, but a tumor with a pseudo-capsule was found on follow-up. The patient was eventually diagnosed using position emission tomography (PET)-CT and biopsy. This case report aims to demonstrate the diagnostic challenges encountered in primary peritoneal cancer, which mimicked sclerosing mesenteritis on CT.

## Case presentation

A 76-year-old woman developed acute-onset right lower abdominal pain and presented to the emergency department. Her medical history included hypertension, hepatitis B, and nodular goiter. She had undergone surgery for appendicitis when she was a teenager. Ten years ago, she underwent surgery and chemotherapy for left lung cancer. On the night before her admission, she had acute abdominal pain. The pain was vague and persistent, hindered her sleep, and worsened with body movement. Based on the review of the systems, she had no nausea, vomiting, diarrhea, chills, fever, or night sweats.

On admission, she had a blood pressure of 154/91 mmHg, pulse rate of 97 beats per minute, body temperature of 37.0°C, and respiratory rate of 16 breaths per minute (SpO_2_, 99% at room air). On physical examination, she had a stiff abdomen with rebound pain noted in the right lower quadrant. Laboratory data showed a white blood cell count of 8,200/μL, erythrocyte sedimentation rate of 58 mm/hour, and C-reactive protein level of 1.40 mg/dL on admission (Table 1).

**Table 1:** Initial laboratory data. PT-INR: prothrombin time-international normalized ratio; APTT: activated partial thromboplastin time

Marker	Level	Reference range
White blood cells	8,200	3.5–9.1 × 10^3^/μL
Neutrophils	60.5	44.0–72.0%
Lymphocytes	27.6	18.0–59.0%
Monocytes	8.7	0.0–12.0%
Eosinophils	2.3	0.0–10.0%
Basophils	0.9	0.0–3.0%
Red blood cells	4.39 × 10^6^	3.76–5.50 × 10^6^/μL
Hemoglobin	14.1	11.3–15.2 g/dL
Hematocrit	41.2	33.4–44.9%
Mean corpuscular volume	93.9	79.0–100.0 fL
Platelets	32.9 × 10^4^	13.0–36.9 × 10^4^/μL
PT-INR	0.88	-
APTT	28.2	25–40 seconds
Erythrocyte sedimentation rate	58	2–10 mm/hour
Total protein	7.3	6.5–8.3 g/dL
Albumin	4.3	3.8–5.3 g/dL
Total bilirubin	0.5	0.2–1.2 mg/dL
Direct bilirubin	0.1	0–0.4 mg/dL
Aspartate aminotransferase	19	8–38 IU/L
Alanine aminotransferase	22	4–43 IU/L
Alkaline phosphatase	95	106–322 U/L
γ-Glutamyl transpeptidase	40	<48 IU/L
Lactate dehydrogenase	182	121–245 U/L
Blood urea nitrogen	14.5	8–20 mg/dL
Creatinine	0.72	0.40–1.10 mg/dL
Serum Na	142	135–150 mEq/L
Serum K	4.2	3.5–5.3 mEq/L
Serum Cl	106	98–110 mEq/L
Serum Ca	9.1	3.5–10.2 mg/dL
Creatine kinase	82	56–244 U/L
C-reactive protein	1.40	<0.30 mg/dL
Thyroid-stimulating hormone	1.55	0.35–4.94 μIU/mL
Free T4	1.1	0.70–1.48 ng/dL
Immunoglobulin G4	22	<135 mg/dL
Urine test		
Leucocytes	(-)	
Nitrite	(-)	
Protein	(-)	
Glucose	(-)	
Urobilinogen	(-)	
Bilirubin	(-)	
Ketone	(-)	
Blood	(-)	
pH	7.5	
Specific gravity	1.033	
Fecal occult blood	Negative	
Anti-nuclear antibody	160	
Homogeneous	(-)	
Speckled	(-)	
Nucleolar	(-)	
Peripheral	(-)	
Discrete	160	
Cytoplasm	(-)	
Proteinase3-anti-neutrophil cytoplasmic antibody	<1.0	U/mL
Myeloperoxidase-anti-neutrophil cytoplasmic antibody	<1.0	U/mL
Anti-SS-A antibody	<1.0	U/mL
Anti-SS-B antibody	<1.0	U/mL
Anti-ds-DNA IgG antibody	<10	IU/mL
Anti-centromere antibody	32.6	U/mL
T-SPOT	(-)	

**Figure 1:**
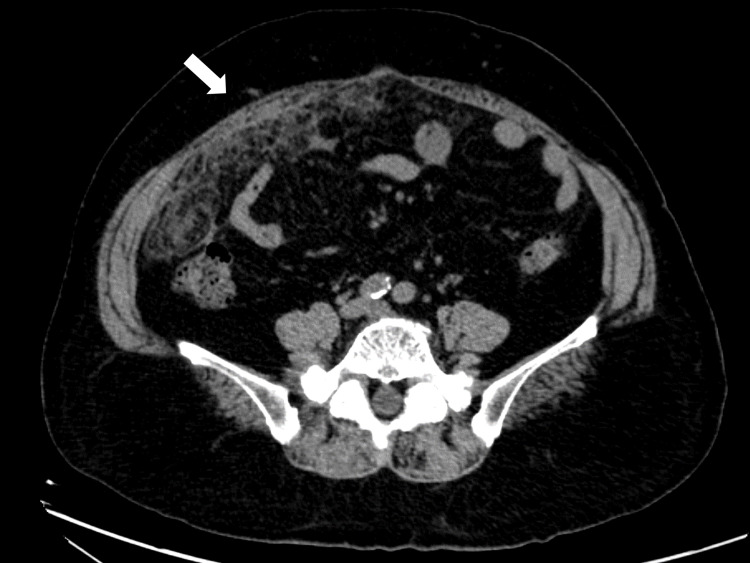
Initial abdominal CT. The image shows diffuse enhancement on the right lower peritoneum. CT: computed tomography

On abdominal CT, diffuse sclerosis and misty appearance of the peritoneum were noted from the right lower quadrant to the pelvic region without any lymphadenopathy (Figure 1).

**Figure 2:**
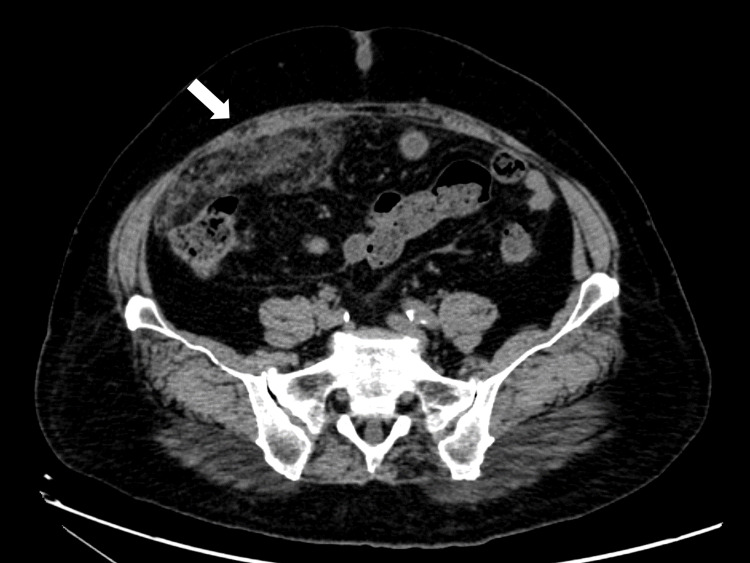
Follow-up CT. The image shows enhancement of the right lower peritoneum mass. CT: computed tomography

Based on the clinical findings, sclerosing mesenteritis was suspected. The patient was prescribed prednisolone (40 mg) and tamoxifen. The abdominal pain improved. A follow-up CT revealed a mass in the right lower quadrant (Figure 2).

**Figure 3:**
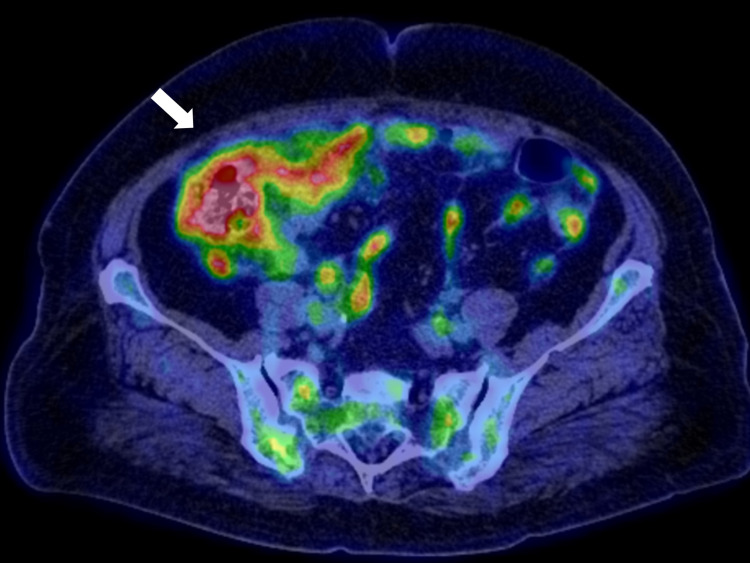
PET imaging of the abdomen. The image shows diffuse high-intensity lesions on the peritoneum, centering on the right lower quadrant. PET: positron emission tomography

She had a cancer antigen 125 (CA-125) of 263.1 U/mL, suggestive of peritoneal cancer. On suspicion of peritoneal cancer, she was referred to the surgery department for an exploratory peritoneal biopsy. A PET scan was performed to detect the biopsy site (Figure 3).

**Figure 4:**
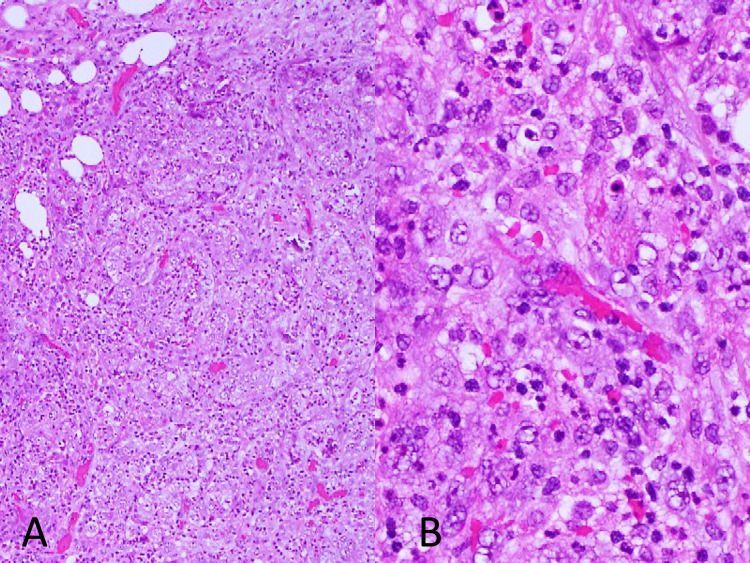
Hematoxylin and eosin stain of the peritoneal tissues. A: original magnification 40×; B: original magnification 400×.

The biopsy revealed a carcinoma without a specific origin (Figure 4).

She was diagnosed with primary peritoneal cancer and referred to the gynecology department. She was treated with N-acetylcysteine following interval debulking surgery at a university hospital. Her symptoms improved and were followed by the gynecologist group in the university hospital.

## Discussion

This case emphasized that acute peritoneal inflammation has various presentations. Thus, it should be differentiated from acute inflammatory diseases and malignancies. In addition, the CT findings of peritoneal inflammation should be interpreted based on the clinical course of the disease. PET, exploratory laparoscopy, and biopsy are also useful in confirming the diagnosis.

In a patient with peritoneal inflammation, differentiating between underlying inflammatory disease and malignancy is challenging owing to the lack of significant clinical findings. The differentiation can be performed based on the onset and time course of symptoms. The onset can be used to differentiate between sclerosing mesenteritis and peritoneal cancer. Sclerosing mesenteritis is the primary inflammation of the peritoneum, involving acute abdominal pain not accompanying other symptoms. In contrast, peritoneal cancer can progress gradually owing to its malignant nature. During progression, this cancer can be accompanied by various symptoms. In this case, the patient presented with acute abdominal pain, which might have involved clinical findings such as mesenteritis.

Initial CT findings can help differentiate the two diseases. Findings of abnormal mesentery and ascites suggest peritoneal cancer [6], which is characterized by abdominal fullness, decreased appetite, and gradual weight loss. In most cases, ascites are detected at the time of diagnosis [6,10]. However, sclerosing mesenteritis can show inflammation of mesenteries with ascites, while the initial presentation of the disease on CT can be inflammation of the mesenteries alone [11]. Therefore, the presence of ascites and diffuse progression on mesenteries can aid in differentiating the diseases. However, neither ascites nor masses were detected on the abdominal CT scan in our case. The present case exhibited atypical imaging findings for a case of peritoneal cancer.

The clinical follow-up by symptoms and CT, leading to further investigation, is critical. It is difficult to differentiate between peritoneal cancer and sclerosing mesenteritis during the early stages. To diagnose the disease, close follow-up is needed to detect abnormalities suggestive of malignancy. Because exploratory laparoscopy is invasive, the patient’s symptoms and physical examination findings should be alarming [9].

A CT scan helps evaluate the status of the disease on follow-up. In this study, the patient’s CT scan findings changed from diffuse inflammation of the mesentery to a localized mass effect in the mesentery after one month. According to a previous study, mass effects on CT imaging suggest peritoneal cancer [12]. The change in imaging findings can indicate the timing for exploratory laparoscopy and biopsy [6,12]. To decide the timing of the biopsy, a change in the quality and quantity of abdominal pain with a corresponding physical examination can be vital.

Furthermore, tumor markers can be useful for diagnosing cancer [13]. In this case, CA-125 was an indicator to proceed with the diagnostic processes of PET and exploratory biopsy [14]. A significantly elevated CA-125 level helps diagnose peritoneal cancer [14].

This study was limited by the insufficient evidence regarding the timing of exploratory laparoscopy and biopsy when differentiating between sclerosing mesenteritis and peritoneal cancer. Our patient initially presented with acute abdominal pain and diffuse inflammation on CT, which suggested sclerosing mesenteritis. However, the following CT findings showed focal inflammation and mass effects in the peritoneum, indicating a malignancy. An exploratory biopsy is invasive and requires general anesthesiology. Thus, physicians should closely follow the clinical findings of patients with sclerosing mesenteritis to avoid missing the diagnosis of peritoneal cancer.

## Conclusions

Peritoneal cancer is difficult to diagnose in its early stage because it resembles sclerosing mesenteritis. Close follow-up to check the patient’s symptoms and CT findings are essential to determine the timing of further investigation with PET and exploratory laparoscopic biopsy.
